# *Trichosporon inkin* empyema and fungaemia following bilateral lung transplantation: diagnostic and treatment challenges

**DOI:** 10.1016/j.mmcr.2026.100806

**Published:** 2026-06-06

**Authors:** David M. Mannion, Assumpta Killarney, Nicola Ronan, Peter Riddell, Breda Lynch, Margaret M. Hannan

**Affiliations:** a Department of Microbiology, Mater Misericordiae University Hospital, Eccles St., Dublin 7, Ireland; b Department of Lung Transplantation, Mater Misericordiae University Hospital, Eccles St., Dublin 7, Ireland; c School of Medicine, University College Dublin, Belfield, Dublin 4, Ireland

## Abstract

A 60-year-old man deteriorated six weeks post bilateral lung transplantation and found to have concurrent *Trichosporon inkin* empyema and fungaemia. Diagnosis was confirmed via biochemical testing and pan-fungal rDNA sequencing. Management involved Interventional Radiology-guided drainage and voriconazole therapy, optimised via therapeutic drug monitoring with a final trough of 1.72 mg/L. This first reported case of concurrent *T. inkin* pleural and bloodstream infection underscores the necessity of molecular diagnostics and aggressive source control.

## 1 Introduction

Invasive fungal infections (IFIs) are a major source of morbidity and mortality among solid organ transplant (SOT) recipients, particularly following lung transplantation, where direct environmental exposure, impaired mucociliary clearance, and prolonged immunosuppression confer a uniquely high susceptibility to opportunistic fungi [[1], [2], [3]]. The International Society for Heart and Lung Transplantation (ISHLT) defines fungal infection in transplant recipients according to standardised criteria [4], and some centres use antifungal prophylaxis in the early post-transplant period to mitigate this risk [1]. Despite these strategies, the spectrum of fungal pathogens has broadened beyond *Candida* and *Aspergillus*, the dominant organisms in most series [5,6].

*Trichosporon* species are basidiomycetous yeasts that typically colonise the skin, gastrointestinal tract, and respiratory airways, but may cause invasive disease in immunocompromised hosts, including those with hematologic malignancy or receiving solid organ transplants [7,8]. Although uncommon, *T. inkin* has emerged as a clinically significant opportunist in transplant recipients, often in the early post-operative period [9,10].

In a recent single-centre study from Spain, *T. inkin* infection was identified in six lung transplant recipients presenting a median of 53 days post-transplantation. Half of these infections were localised (empyema or surgical-site infection), while the remainder were disseminated, with mortality reaching 66% in those with fungaemia [10]. Similarly, Almeida et al. described two cases of *T. inkin* infection (one lung and one heart transplant), both confirmed by intergenic spacer 1 (IGS1) ribosomal DNA sequencing, and highlighted the importance of molecular identification in these complex infections [11].

The therapeutic management of trichosporonosis poses difficulty due to the organism's intrinsic resistance to echinocandins and variable susceptibility to amphotericin B [12]. Azoles, particularly voriconazole, are consistently reported as the most active agents in vitro and in vivo [[11], [12], [13]].

Here, we describe what we believe to be the first reported case of *T. inkin* empyema, with concurrent fungaemia, in a bilateral lung transplant recipient. The case underscores the organism's pleural tropism, the diagnostic pitfalls arising from cross-reactive fungal biomarkers and the critical role of early species identification and azole-based therapy in achieving a favourable clinical outcome. Informed consent to publish was obtained from the patient.

## 2 Case presentation

A 60-year-old man with end-stage hypersensitivity pneumonitis (HSP) was referred for bilateral lung transplantation. His past medical history included ischaemic heart disease. Pre-transplant microbiological screening revealed *Serratia marcescens* in sputum, for which he received a course of piperacillin-tazobactam, and virological testing showed past exposure to herpes simplex virus, varicella zoster virus, and *Toxoplasma gondii* (IgG positive). He was CMV seronegative.

The patient underwent bilateral lung transplantation in 2025**,** using a CMV-seropositive donor. The donor had a history of latent tuberculosis infection, identified during biologic therapy work-up for psoriasis; and only received 3 months treatment pre organ donation. Consequently, isoniazid prophylaxis with pyridoxine was commenced postoperatively in the recipient. Standard immunosuppressive induction and maintenance therapy were instituted with tacrolimus (1.5mg *mane*, 1mg *nocte*), mycophenolate mofetil (1g twice daily), and prednisone (10mg daily). Postoperative antibacterial prophylaxis consisted of meropenem and flucloxacillin for 14 days, with ongoing co-trimoxazole and valganciclovir for *Pneumocystis jirovecii* and CMV prophylaxis, respectively.

The immediate post-transplant period was uncomplicated apart from a transient ischaemic attack (TIA), which necessitated the commencement of high-dose statin therapy. However, by postoperative day 19**,** mild transaminitis developed, attributed to a drug interaction between isoniazid and the statin. In view of the low risk of donor-derived tuberculosis, isoniazid was discontinued, and surveillance bronchoscopy cultures were planned.

**Fig. 1:**
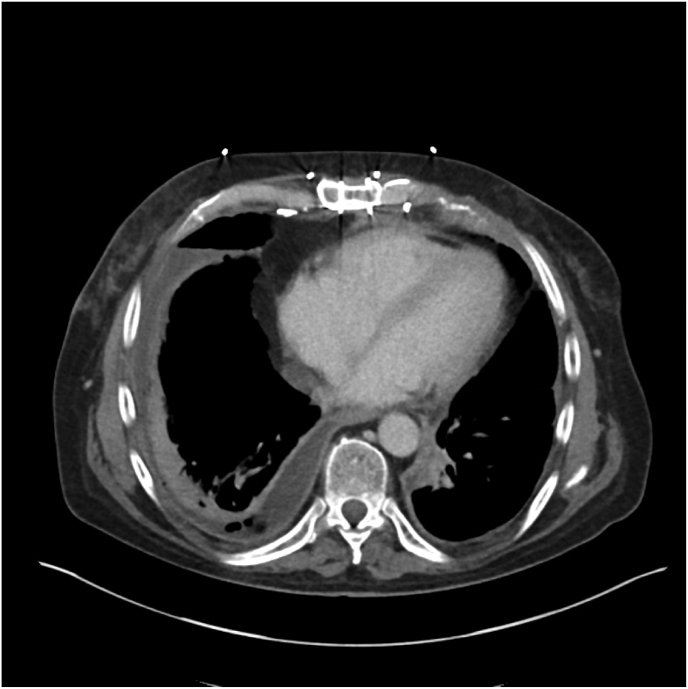
CT-thorax with contrast showing a moderate, right-sided, rim-enhancing hydropneumothorax.

At six weeks post-transplant, while still an in-patient**,** the patient developed progressive dyspnoea, a new oxygen requirement of 2L/min, low-grade fever, and new-onset atrial fibrillation (day 0). Chest imaging demonstrated new bilateral pleural effusions, more pronounced on the right. Empirical therapy with intravenous piperacillin–tazobactam was initiated for presumed bacterial infection. A computed tomography (CT) scan revealed loculated, rim-enhancing hydropneumothoraces bilaterally, associated with small mediastinal and chest wall collections (Fig. 1). Ultrasound-guided drainage of the left pleural effusion on day 1 yielded exudative, haemorrhagic fluid.

### 2.1 Laboratory diagnosis

Initial Gram stain of the pleural fluid showed no organisms seen but a sample that was inoculated into blood culture bottles became positive for yeast cells on day 3. In view of the possibility of a fungal empyema, voriconazole therapy was commenced, chosen for its superior pleural penetration. Cytological examination of the pleural aspirate showed inflammatory cells with septate fungal elements on Grocott methenamine silver (GMS) staining, consistent with a filamentous or pseudohyphal organism.

The patient's antimicrobial cover was then broadened to ceftazidime/avibactam on day 4, due to colonisation with an Oxa-48-producing *C. freundii*, in addition to vancomycin and metronidazole due to further clinical deterioration and ongoing fevers.

**Fig. 2:**
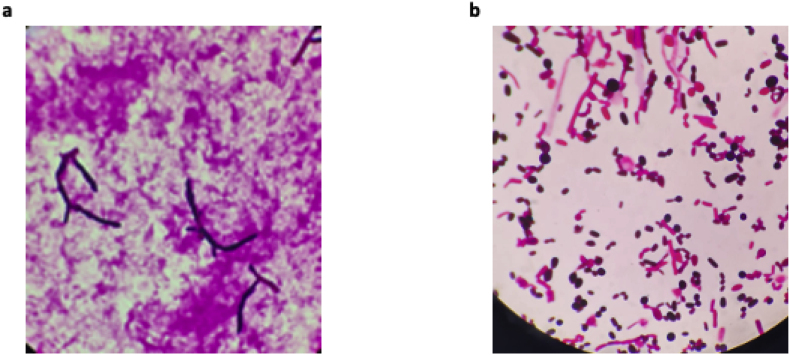
***a.*** Gram-stained blood culture showing septate pseudohyphae consistent with *Trichosporon inkin* (x100). ***b***. Gram stain from blood agar plate showing yeast cells and short pseudohyphae of *Trichosporon inkin* (×100).

**Fig. 3:**
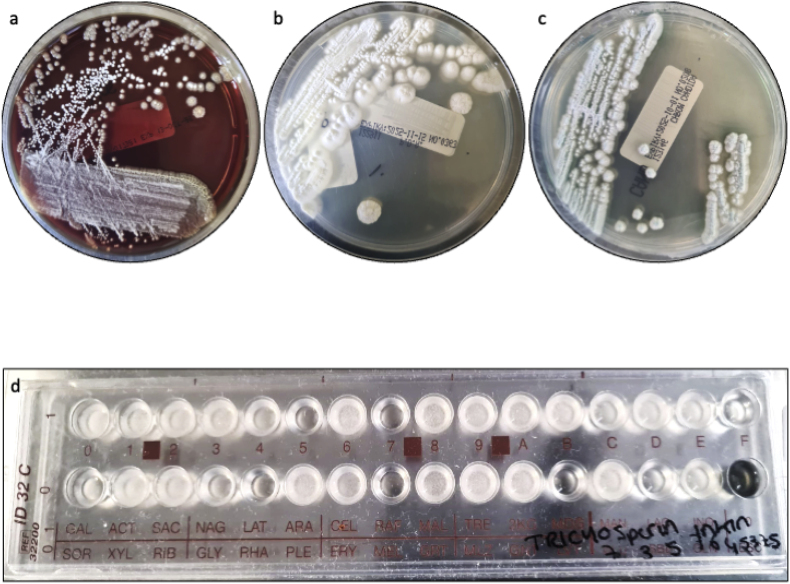
***a.*** Blood agar showing dry, cerebriform, white colonies of *Trichosporon inkin* after 48h incubation at 35 °C. ***b.*** Potato dextrose agar culture demonstrating raised, velvety colonies with radial folding characteristic of *Trichosporon inkin*. ***c.*** Colonies on chromogenic Candida agar appearing pale and non-chromogenic, distinguishing *Trichosporon inkin* from *Candida albicans*. ***d.*** API ID 32C carbohydrate assimilation profile confirming *Trichosporon inkin.*

Blood cultures drawn from both the peripherally inserted central catheter (PICC) and a peripheral vein showed septate pseudohyphae on gram stain (Fig. 2) on day 5 of the acute deterioration. Subsequent culture grew yeast-like colonies and caspofungin was added to the antimicrobial regimen. The isolates were identified as *Trichosporon ovoides* by MALDI-TOF MS (50% confidence). Cryptococcal antigen (CrAg) testing performed concurrently returned positive, at a titre of 1:160, prompting concern for disseminated cryptococcosis however, the patient was neurologically intact and afebrile on clinical review. Given known cross-reactivity between *Trichosporon* and cryptococcal antigen assays, the positive result was considered spurious. The PICC line was removed, and repeat blood cultures were sterile. Biochemical identification was carried out with the API ID 32C yeast identification system (BioMérieux, Marcy-l’Étoile, France) and yielded a result consistent with *T. inkin* (Fig. 3d).

Further molecular analysis of the pleural fluid performed at Great Ormond Street Hospital for Children (GOSH), London, identified *Trichosporon inkin* through pan-fungal PCR targeting the internal transcribed spacer 2 (ITS2) region, followed by sequencing. A parallel specimen was sent to UKHSA Mycology Reference Laboratory, Bristol where species-level identification was confirmed and antifungal susceptibility testing performed. Antifungal susceptibility testing, by broth microdilution method, showed minimum inhibitory concentrations (MIC) of amphotericin B 0.5mg/L, fluconazole 0.5mg/L, and voriconazole <0.03mg/L. Caspofungin was discontinued following confirmation of the organism's identity and expected echinocandin resistance.

### 2.2 Clinical course and treatment

The patient underwent Interventional Radiology–guided drainage of the residual pleural collection on day 30 and repeat cultures were negative for *T. inkin*. He was markedly fluid overloaded during the postoperative period but improved following aggressive diuresis. Transthoracic echocardiography demonstrated preserved biventricular function with no significant cardiac abnormality. CT Abdomen and pelvis and MRI brain did not identify any abnormality. Cardiothoracic surgical review did not identify any evidence of clamshell incision infection. Voriconazole levels were initially subtherapeutic and the dose was increased to 250 mg mane and 300 mg *tarde* orally, achieving a therapeutic trough of 1.72 mg/L at discharge. Repeat blood cultures were sterile. He was reviewed by ophthalmology for visual symptoms, with no evidence of fungal involvement. At discharge, on day 45, he remained clinically stable, with normalisation of inflammatory markers. He was discharged on oral voriconazole with weekly therapeutic drug monitoring, follow-up chest imaging and long-term azole therapy planned for several years to lifelong depending on tolerance and relapse risk.

## 3 Discussion

*Trichosporon* species are increasingly recognised as opportunistic pathogens in SOT recipients, although their incidence remains low relative to *Candida* and *Aspergillus* infections. In the large multicentre TRANSNET cohort, *Trichosporon* spp. accounted for a negligible proportion of invasive fungal infections, underscoring their rarity among SOT recipients [5]. More recent experience from a Spanish lung-transplant centre identified *T. inkin* infection in six recipients with half presenting as empyema or surgical-site infection and the remainder with disseminated disease, among whom mortality reached 66% [10]. These data suggest that while uncommon, *T. inkin* represents an emerging pathogen in the post-transplant setting and appears to exhibit tropism for the pleural space. To our knowledge, this represents the first documented case of concurrent *T. inkin* empyema and fungaemia in a lung-transplant recipient. While previous reports have described either localized pleural infection or disseminated disease, no prior case has demonstrated both simultaneously. This expands the known clinical spectrum of *Trichosporon* infection and highlights its potential for pleural invasion and hematogenous dissemination. Similar findings were observed in a large multicentre study of post-cardiac surgery fungal mediastinitis, where *Trichosporon* spp. accounted for 13% of cases and were associated with 80% mortality, further emphasising the poor outcomes linked to this genus [14].

Our patient developed infection within six weeks of transplantation, consistent with previously reported early postoperative onset [10]. Risk factors commonly described for invasive trichosporonosis include broad-spectrum antibacterial use, central venous catheterisation, calcineurin-inhibitor use, and critical illness [7,13]. In this case, tacrolimus levels were monitored and remained within the therapeutic range, and the patient was not neutropenic at any stage, suggesting that profound immunosuppression or cytopaenia were not principal contributors. The donor lung CT, prior to explantation, demonstrated bilateral consolidation attributed to aspiration rather than fungal infection, and *T. inkin* was not isolated from donor samples, making donor-derived transmission unlikely. Environmental acquisition or early postoperative colonisation followed by pleural invasion appear more plausible.

The diagnosis of trichosporonosis remains challenging owing to cross-reactivity of cryptococcal antigen (CrAg) and Aspergillus galactomannan assays with *Trichosporon* antigens, which may lead to diagnostic delay [10]. In this case, a positive CrAg result was considered spurious, consistent with previously reported cross-reactivity [6].

The pleural isolate and repeat blood cultures confirmed *T. inkin* with minimum inhibitory concentrations of voriconazole <0.03mg/L, supporting its choice as first-line therapy. These results align with published susceptibility data demonstrating intrinsic resistance of *Trichosporon* spp. to echinocandins and variable or reduced susceptibility to amphotericin B, while azoles display the most consistent in-vitro activity and superior clinical outcomes in both disseminated and localized infections [[11], [12], [13],^15^,^16^]. This is an important distinction as guidelines often recommend echinocandins as empiric therapy for invasive fungal infections [14].

In this patient, intravenous voriconazole achieved rapid clinical improvement when combined with drainage of the infected pleural collections. The observed therapeutic voriconazole levels are important, given the variability in azole pharmacokinetics in transplant recipients and the potential for drug–drug interactions with immunosuppressants; however, tacrolimus concentrations remained stable throughout therapy, consistent with careful dose monitoring.

Pharmacokinetic studies demonstrate that azoles, including voriconazole, achieve excellent penetration into pulmonary and pleural compartments, in contrast amphotericin B and echinocandins, which achieve markedly lower pleural concentrations, often below the MIC for most filamentous fungi [16]. These pharmacokinetic data support the selection of voriconazole as the preferred agent for pleural or pulmonary *Trichosporon* infections.

There are no randomized studies or guidelines addressing optimal treatment duration for trichosporonosis in SOT recipients. Reported cases describe treatment courses ranging from several weeks to many months, often continuing azole therapy until complete clinical, microbiological, and radiological resolution [[10], [11], [12]]. Given the organism's potential for persistence and the patient's ongoing immunosuppression, indefinite suppressive voriconazole therapy with therapeutic drug monitoring represents a pragmatic strategy to minimize relapse risk, although such an approach is not yet supported by controlled evidence.

Our patient achieved an excellent outcome, in contrast to other studies showing high mortality [14]. High mortality in other centres may arise from cases where cultures are sterile, possibly from empiric antifungal therapy. In those cases, empiric changes to echinocandins and or Amphotericin B may result in less efficacious therapy. Furthermore, the cross reactivity with CrAG and/or galactomannan could further result in diagnostic and treatment uncertainty in the absence of positive cultures. Therefore awareness of this emerging fungal pathogen is important amongst clinicians managing post lung transplant patients.

## Declarations

No funding was received to assist with the preparation of this manuscript.

## CRediT authorship contribution statement

**David M. Mannion:** Conceptualization, Investigation, Visualization, Writing – original draft. **Assumpta Killarney:** Investigation, Writing – review & editing. **Nicola Ronan:** Investigation, Resources, Writing – review & editing. **Peter Riddell:** Investigation, Resources, Writing – review & editing. **Breda Lynch:** Conceptualization, Investigation, Methodology, Writing – review & editing. **Margaret M. Hannan:** Conceptualization, Investigation, Methodology, Writing – review & editing.

## Conflicts of interest

The authors have no financial or non-financial interests to disclose.
